# Soluble and Endogenous Secretory Receptors for Advanced Glycation End Products in Threatened Preterm Labor and Preterm Premature Rupture of Fetal Membranes

**DOI:** 10.1155/2015/568042

**Published:** 2015-08-27

**Authors:** Rafał Rzepka, Barbara Dołegowska, Aleksandra Rajewska, Sebastian Kwiatkowski, Daria Sałata, Marta Budkowska, Leszek Domański, Wioletta Mikołajek-Bedner, Andrzej Torbé

**Affiliations:** ^1^Department of Obstetrics and Gynecology, Pomeranian Medical University, 70-204 Szczecin, Poland; ^2^Department of Laboratory Diagnostics and Molecular Medicine, Pomeranian Medical University, 70-204 Szczecin, Poland; ^3^Department of Nephrology, Transplantology and Internal Medicine, Pomeranian Medical University, 70-204 Szczecin, Poland

## Abstract

The aim of the study was to compare sRAGE and esRAGE plasma levels in pregnant women with (A) threatened premature labor (*n* = 41), (B) preterm premature rupture of membranes (*n* = 49), and (C) preterm rupture of membranes at term (*n* = 48). The relationship between these and classic intrauterine infection markers and the latent time from symptoms up to delivery depending on RAGE's concentration were investigated. In groups A and B, a positive correlation was found between plasma sRAGE and latent time (*r* = 0,422; *p* = 0,001; *r* = 0,413, *p* = 0,004, resp.). High prognostic values were found in both groups for plasma sRAGE concentration and the latent time from symptoms up to delivery. Groups B and C presented higher levels of esRAGE than group A (526,315 ± 129,453 pg/mL and 576,212 ± 136,237 pg/mL versus 485,918 ± 133,127 pg/mL, *p*< 0,05). The conclusion is that sRAGE concentration can be a favorable prognostic factor in the presence of symptoms of threatened premature labor. Higher esRAGE plasma level in case of the rupture of membranes in mature and premature pregnancy suggests its participation in fetal membranes destruction.

## 1. Introduction 

Preterm labor is defined as a birth of a newborn that occurs between 22nd and 37th week of gestation. Some 30–35% of all premature labors are iatrogenic, due to maternal or fetal indications, while the other 40–65% complete spontaneously in the consequence of preterm uterine contractility or membrane rupture [1]. Preterm end of pregnancy is the most common cause of morbidity and mortality of the newborns as well in the United States as in Europe [1, 2]. Despite the significant development of perinatal medicine in recent years, the prevalence of premature birth has not decreased and it still remains 10–20%. Many factors have been hypothesized as pathogenic for premature labor, but the activation of maternofetal inflammatory response, leading to uterine activity or preterm premature rupture of membranes (pPROM), is believed to be the most corresponding to contemporary knowledge [3].

Most of investigators consider preterm labor as an acute obstetric disease related to ascending bacterial infection of lower pole of membranes with exogenic or endogenic microbes, with subsequent rapid maternal and fetal immunologic response [4–7]. In women diagnosed with chorioamnionitis and premature labor increased plasma levels of some pathogen-associated molecular patterns (PAMPs), such as interleukin-1 beta (IL-1*β*), calcium binding protein A5 (S100A5), prolyl 4-hydroxylase alpha polypeptide 2 (P4HA2), interleukin-6 (IL-6), interleukin-8 (IL-8), lipopolysaccharides (LPS), tumor necrosis factor-alpha (TNF-*α*), and C-reactive protein (CRP), were discovered [8–11]. Yet, in some cases of high PAMPs plasma level in the pregnant, there were no observable symptoms of preterm labor. For this reason present studies on preterm labor pathogenesis focus on the evaluation of chronic inflammation as the predisposing factor for the occurrence of acute intrauterine infection with preterm birth as an outcome.

In 2007 Oppenheim et al. postulated an introduction of the new concept of alarmines to medical nomenclature [12]. Intracellular alarmines, also called damage-associated molecular patterns (DAMPs), can influence immunologic response via toll-like receptors (TLR) and receptors for advanced glycation end products (RAGE) as well as by direct activation of cytokines synthesis in neutrophils and macrophages [13]. The group of the most thoroughly investigated alarmines includes high-mobility group box-1 (HMGB-1), heat shock proteins (HSP), with HSP70 particularly among them, S100A5 protein, hepatoma-derived growth factor (hdgf), IL-1*α*, and uric acid [14–17]. The DAMPs effect is connected with the activation of nonspecific receptors, mainly RAGEs and TLRs. The interaction between RAGE and its ligand yields intensification of oxidative stress not only via NADPH oxidase activation, but also via some transcription activating factors such as nuclear factor kappa-B (NF*κ*B) and mitogen-activated protein kinase (MAPK) [18]. After its release, activated NF*κ*B reaches the nucleus to turn on the expression of genes of cytokines like tumor necrosis factor *α* (TNF*α*), IL-1, and IL-6 and those of adhesive proteins, with vascular cell adhesive molecule-1 (VCAM-1) and intercellular adhesive molecule-1 (ICAM-1) among them, which participate in the inflammatory process [19]. The RAGE-mediated inflammatory response can be modulated by specific negative forms of these receptors, including dominant negative receptors for advanced glycation end products (dsRAGE) and, present in circulating blood, soluble receptors for advanced glycation end products (sRAGE) as well as endogenous secretory receptors for advanced glycation end products (es-RAGE).

High levels of sRAGE are proven to reduce systemic inflammatory response, improving the course and the prognosis in some diseases involving endogenous inflammation [20–25].

From the hypothesis claiming protective effect of negative RAGE forms the question emerges whether the concentration of soluble forms of the aforementioned receptors in the pregnancy can have an impact on the prevalence of preterm labor as a consequence of spontaneous uterine contractility or preterm premature rupture of membranes.

## 2. Objectives

The objectives of this study are as follows:

1. calculation and comparison of sRAGE and esRAGE plasma levels in three groups of pregnant women diagnosed with threatened premature labor, preterm premature rupture of membranes, and preterm rupture of membranes at term,
2. assessment of the relationship between negative RAGE forms and classic intrauterine infection markers,
3. evaluation of pregnancy duration from the occurrence of threatened premature labor symptoms up to delivery, depending on RAGE concentration,
4. evaluation of pregnancy duration from the rupture of membranes until delivery, depending on RAGE concentration.

## 3. Materials and Methods

The study was conducted in the Department of Obstetrics and Gynecology and in the Department of Laboratory Diagnostics and Molecular Medicine of Pomeranian Medical University from 29/10/2012 to 30/06/2014. 138 women between 22nd and 41st week of gestation were included and subsequently divided into three groups. Group A contained 41 women between 22nd and 36th week of gestation, presenting symptoms of threatened premature labor. Group B enclosed 49 participants between 22nd and 36th week of gestation with preterm premature rupture of membranes. Group C brought together 48 pregnancies with the evidence of preterm rupture of membranes but lacking in uterine contractions, who completed 37th week of gestation.

The detailed characteristics of study groups is shown in Table 1 and the inclusion criteria are listed in Table 2.

Not later than two hours after admission to the department, peripheral maternal blood was sampled from the ulnar vein and then treated with dipotassium ethylenediaminetetraacetic acid (EDTA-K_2_). After centrifugation of the whole sample (10 minutes, 5000 rps), obtained plasma samples were stored at −80°C until sRAGE and esRAGE concentration analyses were performed. Immunoassay methods were used to sRAGE and esRAGE calculations. Human sRAGE ELISA (Bio Vendor Research and Diagnostic Products) is a sandwich enzyme immunoassay for quantitative measurement of human sRAGE. Calibration range for sRAGE is 50–3200 pg/mL, with the limit of detection at 19,2 pg/mL. Human esRAGE ELISA (Cusabio, CSB-E15773h) is analogically a sandwich enzyme immunoassay for quantitative measurement of human esRAGE. Calibration range for esRAGE is 0,625 ng/mL–40 ng/mL, with the limit of detection at 0,156 ng/mL. Coefficients of variation (CV) for the assays of sRAGE and esRAGE are shown in Table 3.

All women included in the study had also determined white blood cells count and neutrophils percentage in venous blood and plasma level of C-reactive protein. In the participants in groups A and B the ultrasound cervical length was assessed with a vaginal probe placed in the vestibule of the vagina. The arithmetic mean of the three subsequent measurements was used in the study. Moreover, in every woman belonging to group A or B, a microbiological smear for aerobic bacteria culture was taken from the cervical canal during gynecologic examination. In group A, after exclusion of diagnosis of intrauterine infection, intravenous inflow of Fenoterol in dosage range from 0,0035 to 0,005 mg/min was administered as a tocolytic agent, until the inhibition of uterine contractions. The participants were also given Betamethasone in two 12 mg doses with 24-hour intervals to accelerate fetal lung maturation.

Eventually the whole group A was categorized into subgroups by the duration of pregnancy from the diagnosis of threatened premature labor up to delivery, with a seven day cut-off point.

After diagnosis, antibiotic agents were administered in group B to extend the duration of pregnancy between the rupture of membranes and delivery. Intravenous 2 g of Ampicilin and 300 mg of Erythromycin every six hours for 48 hours and subsequently oral 500 mg of Amoxycilin every eight hours and 250 mg of Erythromycin every six hours for five days were considered as a standard. These women were also given two 12 mg doses of Betamethasone with 24-hour intervals to accelerate fetal lung maturation, avoiding tocolytic agents administration.

Group B was additionally divided into subgroups according to pregnancy duration from the rupture of membranes to delivery, with the cut-off point considered as 24 hours.

The study was approved by the Bioethical Committee of Pomeranian Medical University (KB-0012/121/12).

### 3.1. Statistical Analysis

The statistical evaluation was performed using Statistica 10,0 PL software for Windows. The distribution of variables was checked using nonparametric W Shapiro-Wilk's test and, according to its results, values were further analyzed. The level of significance (*p*) was set at less than 0,05. For the presentation of not normally distributed variables the number of patients (*N*), values' range (min–max), medians (Me), and the first and third quartile values (*Q*1-*Q*3) were included in the descriptive statistics. However, for normally distributed variables' characteristics, the results were presented as number of patients (*N*), arithmetical mean (*X*), and standard deviation (SD). To assess the differences between analyzed parameters between two groups the Mann-Whitney test for unpaired variables was used. We used the ANOVA Kruskall-Wallis test to assess the differences between analyzed parameters between more than two groups. For the statistical analysis of relationship between *X* and *Y*, correlations' coefficients were estimated using Spearman's test. Receiver operation characteristic (ROC) curve analyses to determine the cut-off point, as well as the predictive value of tests, their sensitivity, specificity, positive and negative predictive values (PPV and NPV, resp.), and accuracy, were analysed.

## 4. Results

The majority of studied parameter distributions deviated from the normal distribution (Shapiro-Wilk test *p* > 0,05). Patients did not differ significantly between study groups in terms of age, white blood cell counts, banded neutrophils, CRP plasma level, and sRAGE concentration. There were significant differences in esRAGE levels found between groups.  Table 4 shows the summary of results obtained in each group.

In groups B and C, higher levels of esRAGE were found compared with group A (526,315 ± 129,453 pg/mL and 576,212 ± 136,237 pg/mL versus 485,918 ± 133,127 pg/mL, *p* < 0,05).

Rank correlation was analyzed in A and B groups between the levels of sRAGE and esRAGE and white blood cells count, CRP concentration, banded neutrophils, cervical microbiological smear findings, cervical length in ultrasound examination, neonate birth weight, gestational age at delivery, and latency period after diagnosis. A positive correlation was found in group A between plasma sRAGE concentration and pregnancy duration from diagnosis to delivery (*r* = 0,422; *p* = 0,001), neonate birth weight (*r* = 0,338; *p* = 0,03), and gestational age at delivery (*r* = 0,469; *p* = 0,002). In B group the latency period from diagnosis to delivery seemed to extend in the presence of increasing sRAGE plasma levels (Table 5).

**Figure 1:**
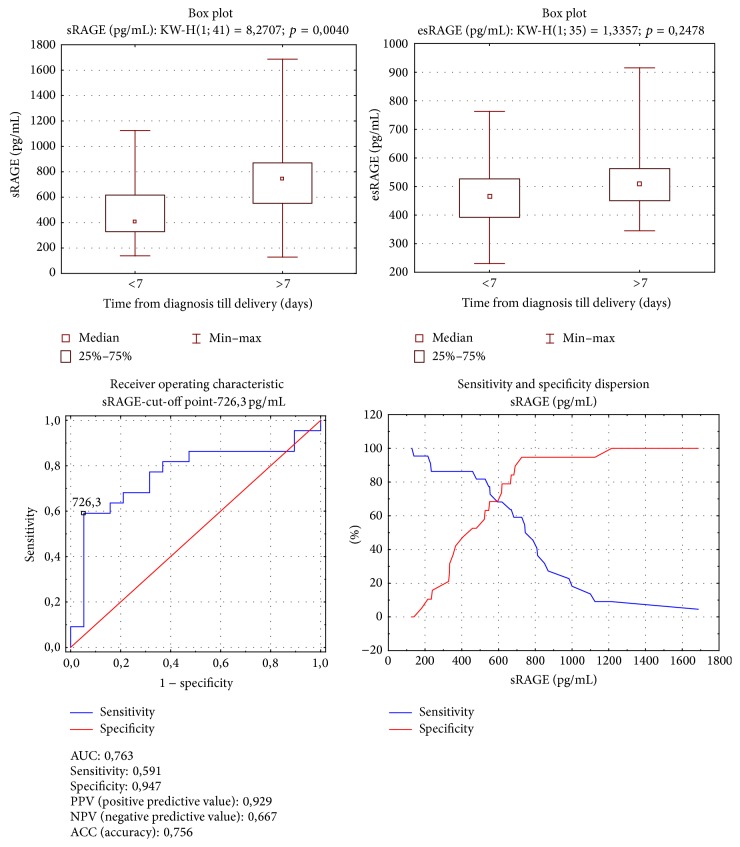
Group A. sRAGE and esRAGE comparison according to 7-day latent time. Mann-Whitney test; sRAGE ROC curve analysis for latent time.

In the subgroup of pregnancies with latency period extended over seven days, significantly higher concentrations of sRAGE were found, compared with those with latency less than seven days (Me = 405,923 versus 744,001 pg/mL; *p* = 0,004). The analysis of area under the ROC curve for sRAGE and latency period revealed the cut-off point for latency duration over seven days as a sRAGE concentration of 726,300 pg/mL, with test specificity 0,947 and sensitivity 0,591 (Figure 1).

**Figure 2:**
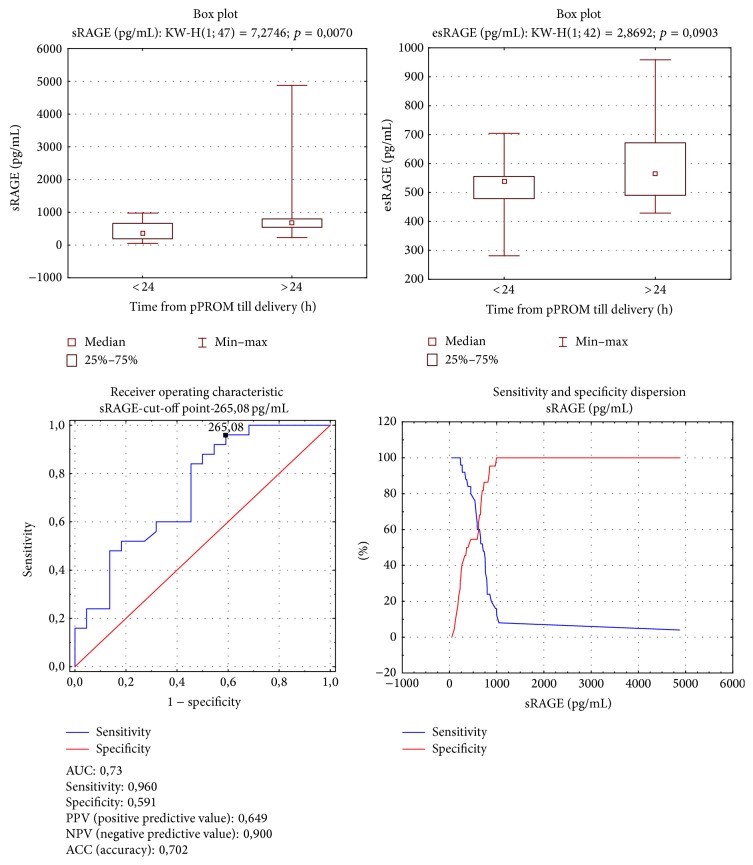
Group B. sRAGE and esRAGE comparison according to 24-hour latent time. Mann-Whitney test. sRAGE ROC curve analysis for latent time.

In group B higher sRAGE levels occurred in women whose latency period from pPROM until delivery was over 24 hours (Me = 712,05 pg/mL versus 368,11 pg/mL; *p* = 0,007). Both subgroups presented comparable esRAGE concentrations. The analysis of area under the ROC curve for sRAGE and latency period in group B yielded an sRAGE level equal to 265,08 pg/mL as the cut-off point for latency period over 24 hours, with test specificity 0,591 and sensitivity 0,960 (Figure 2).

## 5. Discussion

### 5.1. Receptors for Advanced Glycation End Products

The receptors for advanced glycation end products (RAGE) pertain to the group of transmembrane multiligand receptors belonging to the immunoglobulin superfamily, the activation of which is crucial, inter alia, for induction and maintenance of inflammatory response [26–28]. The RAGE is located on the surface of many cell populations, including phagocytes, hepatocytes, endothelium, smooth muscles of blood vessel media, nervous system cells, and mesangial cells of the glomeruli [26]. The RAGE determining gene is localized on the 6th chromosome, next to the major histocompatibility complex class III region [29]. This location makes RAGE probable not only to act as a receptor but also to be involved in the reaction to different types of injury [30]. It has been proven that the RAGE gene expression can be induced in response to enhanced cell activation caused by increased concentration of RAGE ligands in case of tissue damage and inflammation [27].

Apart from the native RAGE form, there are some additional, mostly negative, isoforms described in the literature. Negative RAGEs are heterogenic protein groups, capable of binding, inter alia, advanced glycation end products (AGE) and alarmines. Thanks to ligands binding, negative RAGEs inhibit the response mediated by advanced glycation end products and some alarmines and have a protective effect on blood vessels against toxic influence of ligand-RAGE complexes [31–33]. The membrane form of the receptor, known as dominating negative RAGE (dnRAGE), can be found on the cell surface. The ligands attached to the receptor concentrate on the cell surface, which results in suppression of the receptor's signal transduction [28, 34]. The sRAGE-ligand complexes lose their affinity for heparan sulfate to be released to the blood stream and to be, eventually, captured and degraded in the liver or spleen. A particular type of sRAGE lacking the transmembrane and cytosolic domains is thought to be alternatively spliced and named as the endogenous secretory RAGE (esRAGE) [33].

### 5.2. The RAGEs and Premature Labor

Prior assumption of the protective influence of negative RAGEs leads to the question whether different levels of receptors' soluble forms in the pregnant can correlate with the prevalence of premature labor, considered both are a sequel of spontaneous uterine contractility or premature rupture of membranes as well. Because the main objective of the study was to analyze a potential role of negative RAGEs in the pathogenesis of preterm labor, sRAGE and esRAGE concentration was assessed in all participants and compared among study groups. There is little research on the evaluation of sRAGE and esRAGE levels in pregnant women suffering from threatened preterm labor and/or premature rupture of membranes. Hájek et al. found sRAGE mean concentration in pregnancy to be about 669 ± 296 pg/mL and estimated lower sRAGE concentration in women diagnosed with threatened premature labor than in healthy pregnant women [35]. In our study sRAGE levels were compared reaching, respectively, 594,9, 612,9, and 714,45 pg/mL in groups A, B, and C, without significant differences among the groups, which is in contrast with findings of Hájek et al. [35]. Following the quest, Germanová et al. determined sRAGE levels to be the lowest in the 1st and 3rd trimesters of uncomplicated pregnancy, tending to decrease in women diagnosed with preterm labor and/or preeclampsia [36]. The weak point of the study was the small number of participants, amounting to 42.

In our research the sRAGE concentration was also the lowest the in preterm labor group, but, compared with the other, did not differ significantly, while the analysis and comparison of esRAGE concentration proved remarkable differences among study groups. Women from groups B (pPROM) and C (PROM) had significantly higher levels of esRAGE than those from group A. The question of whether, and in what mechanism, increased concentration of esRAGE is associated with the preterm rupture of membranes should be considered in the near future. The connection between sRAGE and esRAGE plasma levels and the rupture of membranes has not been proven in literature till now, since most of the research concerned the role of amniotic fluid RAGEs concentration in premature labor [37].

Roberto Romero is the leading investigator of RAGEs in the obstetric context. In 2008 he evaluated sRAGE and esRAGE amniotic fluid concentration in categorized groups of the pregnant women: in their 2nd trimester of pregnancy, at term but lacking symptomatic labor, at term in labor, diagnosed with threatened preterm labor with unruptured membranes, and diagnosed with preterm premature rupture of fetal membranes. He assessed receptor concentrations depending on the presence or absence of intrauterine infection in preterm pregnancies, finding that participants with symptoms of labor at term had lower levels of sRAGE, while in women at preterm labor lower sRAGE concentration correlated with intrauterine infection [37]. Taking into account the molecular patterns of RAGEs function in the modulation of inflammatory response, the findings cited above must be regarded as unexpected [38].

Our study focused on calculation of plasma RAGEs concentration only, since we presume that confirmation of association of sRAGE and esRAGE plasma levels with preterm labor would result not only in future research, but above all as an introduction of a new clinical diagnostics tool. The correlation between RAGEs (sRAGE and esRAGE) and other preterm labor markers was evaluated in A and B study groups. The measurement of latency period, from the onset of premature labor symptoms till delivery in group A and from premature rupture of membranes up to delivery in group B, was the particular element of further statistical analysis. The cut-off point was determined at seven days in group A and at 24 hours in group B, which has its practical implication; that is, estimation of latency period in a pregnant women with threatened premature labor for over seven days lets her safely leave the hospital after adequate treatment. There was positive correlation in group A between plasma sRAGE levels and latency period duration, gestational age at delivery, and neonatal birth weight. High values of correlation coefficient for sRAGE plasma concentration and latency (*r* = 0,42) and for sRAGE and gestational age at delivery (*r* = 0,47) became a good reason for analysis of the area under the ROC curve, in order to evaluate the sRAGE plasma level cut-off point at which premature labor occurs later than seven days from the onset of symptoms. We considered sRAGE plasma concentration over 726,3 pg/mL to be the predictive factor of preterm delivery, with sensitivity 0,591 and specificity 0,947. The results obtained in our study seem to be consistent with the protective function of negative forms of RAGEs, which has already been proven and described in the literature [39–41]. The protective effect of increased sRAGE plasma level was also documented by Bastek et al., who analyzed it in the group of pregnant women (*n* = 529) suffering from threatened preterm labor, from which 39,8% of the participants gave premature birth. In the last subgroup significantly lower plasma sRAGE concentration was detected (771,79 pg/mL), compared with those who delivered at term (948,485 pg/mL). The authors concluded that sRAGE plasma level estimation would be useful as a preterm delivery prognostic marker and its high value can be a favorable prognostic factor. They also evaluated cord blood sRAGE concentration in the infants born to participants of the study, finding higher sRAGE levels correlated with lower occurrence of sepsis in the neonates [42]. After the rupture of membranes in preterm gestation, potential risk of overt intrauterine infection is associated with pregnancy prolongation [43, 44]. Increased plasmatic levels of CRP, IL-1, and procalcitonin, as well as observable symptoms, suggest infection development [45, 46]. The diagnosis of overt intrauterine infection is an adverse prognostic factor for premature newborns. From the other point of view, extension of pregnancy duration after preterm rupture of membranes, especially those occurring before 30 weeks of gestation, may increase the chance of proper psychomotor development in preterm infants [47]. There is a requirement of a marker of intrauterine infection onset, indicative of the immediate need for parturition.

In our study group B we analyzed the correlation between RAGEs (sRAGE and esRAGE) plasma levels and latency period from the rupture of membranes till delivery, as well as white blood cell counts, CRP concentration, banded neutrophils percentage, ultrasound cervical length, microbial cervical cultures, gestational age at delivery, and neonatal birth weight. What we found was positive relation between sRAGE concentration and latency period duration and between esRAGE and CRP levels. It can be assumed that a higher sRAGE level in this group reduces the risk of rapid intrauterine infection development. Romero et al. were the only ones in 2012 to publish a scientific report on amniotic fluid sRAGE concentration depending on presence or absence of chorioamnionitis in mature pregnancies. They detected a decrease of sRAGE level accompanying signs and symptoms of intrauterine infection [48]. Although in our study the sRAGE level correlated with duration of latency period after pPROM, it was not associated with WBC, neutrophils percentage in blood smear, or with ultrasound cervical length. We observed a negative trend for sRAGE and CRP concentration (*r* = −0,303; *p* = 0,056). In the literature, only Germanová et al. found strong negative correlation between sRAGE and WBC (*r* = −0,47) [36]. After having found the association between sRAGE and latency period, using the analysis of the area under the ROC curve, we calculated the cut-off point for sRAGE level over which the latency period exceeded 24 hours. The specificity of the test was 0,59 and its sensitivity reached 0,96 for a sRAGE concentration of 265,08 pg/mL. In the literature on this issue lacks the value of sRAGE level indicating rapid intrauterine infection development following preterm premature rupture of membranes. In preterm pregnancy pPROM can be as well a cause as a consequence of chorioamnionitis. It seems that, in case of pPROM following intrauterine infection, the latency period is reduced to less than 24 hours and sRAGE level is low, while in the opposite case the latency exceeds 24 hours, accompanied by significantly higher sRAGE concentration. Unfortunately, only very few researchers are concerned with the evaluation of different forms of RAGEs in premature labor [36, 37, 42, 48]. Positive correlation between esRAGE and CRP seems to prove that every form of RAGE can play a different role in preterm labor pathogenesis. The results obtained in our study, which is partly consistent with the other authors' research, demonstrate the role of DAMPs and many forms of their receptors in the pathogenesis of premature labor. The paucity of the literature on the topic and contradictory results of some studies show the need for future research on the role of RAGEs in preterm labor.

## 6. Conclusions

1. Higher plasma esRAGE concentration in the pregnant women with the rupture of membranes in mature and premature pregnancy suggests its participation in fetal membranes destruction. There is a need for further study on esRAGE in women after PROM.
2. The lack of significant association between white blood cell count or neutrophils percentage and sRAGE level shows that the role of the latter is not necessarily connected with intrauterine infection.
3. High sRAGE concentration can be a favorable prognostic factor in the presence of symptoms of threatened premature labor. There is a need for more research to confirm results of this observation.
4. High sRAGE plasma concentration in premature gestation complicated by preterm rupture of the membranes seems to delay the development of intrauterine infection. More studies on this topic are necessary to prove our observation.

## Figures and Tables

**Table 1:** General characteristics of the studied population (mean ± SD).

Parameters	Group A	Group B	Group C	*p* value
Number of women	41	49	48	—
Age [years]	28 ± 6	30 ± 7	29 ± 6	NS
Gestational age [weeks]	31 ± 3	31 ± 4	39 ± 1	*p* = 0,0001
Parity	2 ± 1	2 ± 1	2 ± 1	NS

**Table 2:** Inclusion criteria.

Group	A (*n* = 41)	B (*n* = 49)	C (*n* = 48)
Week of gestation	22–36	22–36	37–41

Signs & symptoms	Uterine contractions of the frequency ≥4 per 60 minutes, documented in CTG record.	Absence of uterine contractions, documented in CTG records.	Absence of uterine contractions, documented in CTG records.
	Bishop score ≥4		
	Cervix length ≤25 mm documented in ultrasound scans.	Rupture of fetal membranes confirmed with one of the following: (i) vaginal discharge pH ≥7, (ii) positive result of Amni Sure test, (iii) positive result of Amni Prom test.	Rupture of fetal membranes confirmed with one of the following: (i) vaginal discharge pH ≥7, (ii) positive result of Amni Sure test, (iii) positive result of Amni Prom test.

**Table 3:** Coefficients of variation for the assays.

Assays	Coefficients of variation (CV)	
	Intra-assay (within-run) [%]	Interassay (run-to-run) [%]
sRAGE	4.00	7.15
esRAGE	5.20	8.50

**Table 4:** Descriptive statistics of studies groups by ANOVA Kruskall-Wallis comparison.

Parameters	Group A					Group B					Group C					*p*
	*N*	min–max	*Q*1	*Q*3	Me	*N*	min–max	*Q*1	*Q*3	Me	*N*	min–max	*Q*1	*Q*3	Me	
Age [years]	41	15–41	24	32	30	49	16–41	26	35	31	48	17–40	25	33	29	NS
WBC [G/L]	41	3,3–20,0	9,51	14,4	13,19	49	8,2–25,4	10,05	14,38	11,82	48	8,0–21,0	9,97	14,04	11,43	NS
CRP [mg/L]	41	0,4–39,5	2,3	5,5	3,7	49	0,2–77,3	2,7	11,8	5,8	48	0,3–19,0	1,5	5,4	3,7	NS
Band [%]	41	63–92	74,2	79,4	76,8	49	55–91	66,7	80,8	71,7	48	60–91	66,9	79,8	73,0	NS
sRAGE [pg/mL]	41	128–1686	352	787	594	49	48–4872	297	775	612	48	77–1787	285	887	714	NS
esRAGE [pg/mL]	41	230–915	406	533	490	49	281–958	483	610	541	48	410–1096	497	613	542	**0,00**

WBC: white blood cells.

CRP: C-reactive protein serum level.

Band: banded neutrophils.

sRAGE: secretory receptors for advanced glycation end products.

esRAGE: endogenous secretory receptors for advanced glycation end products.

*Q*1: Quartile 1.

*Q*3: Quartile 3.

Me: median.

*p*: level of significance.

**Table 5:** Correlations between sRAGE, esRAGE, and biochemical and clinical markers of preterm labor. Groups A and B (*N* = 89).

Correlations	Group A		Group B		Correlations	Group A		Group B	
	*R*	*p*	*R*	*p*		*R*	*p*	*R*	*p*
esRAGE versus WBC	0,149	NS	0,030	NS	sRAGE versus WBC	0,070	NS	0,022	NS
esRAGE versus CRP	0,238	NS	**0,390**	**0,02**	sRAGE versus CRP	−0,303	NS	−0,304	NS
esRAGE versus Band	0,133	NS	0,035	NS	sRAGE versus Band	−0,171	NS	−0,179	NS
esRAGE versus MC	−0,165	NS	−0,148	NS	sRAGE versus MC	−0,165	NS	−0,177	NS
esRAGE versus CxL	−0,186	NS	0,002	NS	sRAGE versus CxL	0,017	NS	0,002	NS
esRAGE versus LT	0,212	NS	0,185	NS	**sRAGE versusLT**	**0,422**	**0,001**	**0,413**	**0,004**
esRAGE versus GD	0,045	NS	0,069	NS	sRAGE versus GD	0,469	0,002	0,069	NS
esRAGE versus BW	0,038	NS	0,091	NS	sRAGE versus BW	0,338	0,03	0,047	NS

*p*: level of significance.

*R*: Spearman's correlation rate.

sRAGE: secretory receptors for advanced glycation end products.

esRAGE: endogenous secretory receptors for advanced glycation end products.

WBC: white blood cells.

CRP: C-reactive protein.

Band: banded neutrophils.

MC: microbial culture from the cervix.

CxL: ultrasound cervical length.

LT: latent time, from pPROM till delivery and from diagnosis of threatened preterm labour till delivery.

GD: gestational age at delivery.

BW: birth weight.
